# Editorial Brevities

**Published:** 1887-09

**Authors:** 


### EDITORIAL BREVITIES.

DEATH OF DR. WADSWORTH.—We regret to learn of the recent death of Dr. J. N. Wadsworth, of Oglethorpe, Georgia.

THE MEDICAL CONGRESS ABROAD.—In Vienna, Austria, a local committee of twenty-five has been appointed to make arrangements for attending the International Medical Congress.

PROFESSOR J. F. HARRISON has resigned the Chair of Practice in the University of Virginia. We have not learned who is to be his successor.

S. T. YOUNT.—See the interesting advertisement of Yount's Double Slide Rectal Speculum (illustrated), in to day's journal.

THE Introductory Address in the Atlanta Medical College will be delivered by Professor V. H. Taliaferro.

A NUMBER of Georgia physicians will visit the International Medical Congress on the 5th instant. Several will go from Atlanta.

OPIATES ADVANCING.—It is predicted that opiates will advance by reason of failure of the poppy crop in Smyrna, from whence our supplies of that article are largely derived.

THE opening address in the Southern Medical College will be delivered by R. C. Word, M.D., Prof. of Physiology, on Wednesday, October the 5th, at 11 o'clock. The profession and the public generally are invited to be present.

WE had a pleasant call recently from Dr. J. L. Hamilton, the Senator representing the counties of DeKalb, Henry and Gwinnett in the Georgia Legislature.

MORTALITY.—Recent statistics from extensive sources in Europe would seem to indicate that the average rate of mortality in that country is 22.4 per 1,000 of the aggregate population.

DR. ORME'S ADDRESS.—We are in receipt of an address, delivered at the American Institute of Homœopathy, by our fellow citizen Dr. T. H. Orme. The doctor's address is ably gotten up, and we opine makes as fair a showing of the system which he advocates as can be done. He claims that Homœopathy, so far from dying out, is making rapid strides in the United States.

A DIPLOMA DISGRACED.—The Nashville Medical College recently passed a resolution denouncing W. P. Younger, of Kentucky, one of her graduates, for "entering into all manner of advertising schemes to obtain practice in violation of the code, professional decency, etc.," and ordered his name stricken from their records as an alumnus.

EXCURSION TO CALIFORNIA.—Among the advantages to be given the members of the International Medical Congress will be an exceedingly cheap excursion to California. The rate will be less than the fare one way. This will be from Washington only about $90. This offer is extended to both foreign and American members of the Congress.

DR. JAMES A. GRAY thinks that $6,000 a year can be made by boarding, nursing and treating pauper patients at 75 cents per day. This will be good news to numerous physicians who find it hard to live at the ordinary practice. We presume the doctor will soon abandon his present business and establish a pauper hospital.

OUR HOSPITALS.—It is true, as stated by Dr. Gray, that he can substantiate the fact that the Hospitals of the city receive, by contract, a stipulated sum from the Council for treating the poor. This no one denies. It could be shown as easily that he meant no good to the city, or those who are laboring for the benefit of the sick in these Hospitals; but we do not believe that the animus of this article is such as will be indorsed by the "best men in the city," or by the unprejudiced men of the profession.

